# Current drug use patterns and HIV and HCV prevalence among people who inject drugs in suburban areas of Malaysia

**DOI:** 10.1002/jia2.26420

**Published:** 2025-04-25

**Authors:** Joselyn Pang, Mahmoud Danaee, Vicknasingam Balasingam Kasinather, Don Des Jarlais, Adeeba Kamarulzaman, NA Mohd Salleh

**Affiliations:** ^1^ Department of Medicine, Faculty of Medicine University of Malaya Kuala Lumpur Malaysia; ^2^ Department of Social and Preventive Medicine, Faculty of Medicine University of Malaya Kuala Lumpur Malaysia; ^3^ Centre for Drug Research Universiti Sains Malaysia Penang Malaysia; ^4^ New York University School of Global Public Health New York New York USA; ^5^ Monash University Malaysia Selangor Malaysia; ^6^ Centre of Excellence for Research in Infectious Disease and AIDS University of Malaya Kuala Lumpur Malaysia

**Keywords:** amphetamine‐type stimulant, harm reduction, hepatitis C, heroin use, HIV prevention, people who inject drugs

## Abstract

**Introduction:**

National surveillance data in Malaysia has observed a marked reduction in the number of new HIV cases among people who inject drugs (PWID) in the past decade. This study sought to estimate the current prevalence and associated risk factors of HIV and hepatitis C virus (HCV) among PWID in suburban areas of Klang Valley, Malaysia.

**Methods:**

Between September 2021 and March 2022, a cross‐sectional, respondent‐driven sampling survey was conducted. Participants completed rapid HIV and HCV testing as well as social and behavioural assessments. Factors associated with HIV‐ and HCV‐positive results were estimated using logistic regression.

**Results:**

Four‐hundred individuals were recruited in the study, of whom 382 (94%) were men. The prevalence of HIV and HCV was 5.5% (95% confidence interval [95% CI]: 3.6–8.3) and 40.5% (95% CI: 35.7–45.5), respectively. Current heroin and amphetamine‐type stimulant (ATS) use, regardless of injection or non‐injection use, were reported by 340 (85.0%) and 328 (82.0%) individuals, respectively. Past exposure to the criminal justice system (lock‐ups, prison and compulsory drug detention centres) was associated with both HIV (Adjusted odds ratio [aOR] = 3.47, 95% CI: 1.33–10.2) and HCV (aOR = 3.32, 95% CI: 2.06–5.39)‐positive results. Additionally, HIV‐positive results were associated with current ATS use (aOR = 0.31, 95% CI: 0.12–0.86). Meanwhile, HCV‐positive results were associated with current heroin use (aOR = 2.44, 95% CI: 1.16–5.48), lifetime enrolment in methadone treatment (aOR = 2.30, 95% CI: 1.23–4.27), current methadone treatment (aOR = 0.46, 95% CI: 0.23–0.92) and current mixing of drugs through injection use (aOR = 1.80, 95% CI: 1.08–3.03).

**Conclusions:**

This study observed low HIV prevalence among PWID, primarily associated with ATS use, while HCV prevalence, linked to heroin use, remained high. Higher odds of being HCV positive among PWID who reported to have ever but not currently enrolled in methadone programmes indicate that treatment may not be continuous once initiated, potentially due to exposure to the criminal justice system. These findings underscore the need for a dual approach: enhanced harm reduction programmes for PWID and a legal reform to address potential barriers posed by criminalization.

## 1 INTRODUCTION

The HIV epidemic has been classified as a concentrated epidemic in Malaysia, with a high estimated prevalence among people who inject drugs (PWID) due to shared injecting equipment [1, 2]. It is estimated that there are 156,000 PWID in 2017, making up 66% of the total number of people living with HIV in the country [3]. Since its implementation in 2005, harm reduction programmes such as methadone and needle‐syringe services have contributed significantly to the reduction of HIV prevalence [4]. HIV prevalence among PWID has reduced from 22% in 2009 to 13.4% in 2017 [4].

Malaysia has a moderate hepatitis C virus (HCV) burden, with an estimated anti‐HCV prevalence of 1.9% [5]. However, a cross‐sectional study at the end of 2020 across Malaysia found that the weighted prevalence of HCV infection in Malaysia was 0.4% (95% CI 0.2–0.7) amounting to an estimated 90,119 infected population age ≥15 years. More than half of them were chronically infected, with the prevalence of 0.2% (95% CI 0.1–0.4) [6]. Data from the Ministry of Health Malaysia showed 3438 new chronic infections in 2019 and the number increased to 4626 in 2022 [6].

Few studies were conducted previously to determine the prevalence and factors associated with HIV and HCV in Malaysia [7, 8, 9, 10]. Socio‐demographic factors (age, income, education), duration of injecting drug use and sharing needles were factors associated with HIV and HCV positivity results in these studies. However, very few studies have been conducted to examine the association between the legal and social environment and HIV/HCV infections, especially in the current context of increased amphetamine‐type stimulant (ATS) use. The aim of this study was to estimate the current prevalence and associated factors of HIV and HCV among PWID in Klang Valley, Malaysia.

## 2 METHODS

### 2.1 Study design and recruitment

Between September 2021 and March 2022, 400 individuals were recruited in a cross‐sectional, respondent‐driven sampling (RDS) survey. The study aimed to assess socio‐demographic factors; income and material security; homelessness and living conditions; drug use patterns; HIV risk behaviours; experiences with the criminal justice system (CJS); health status and access to support services, including healthcare among PWID in Klang, Selangor. Participants were recruited based on inclusion criteria such as (1) being over 18 years old, (2) history of drug injection, as confirmed by injection marks and knowledge of drug injecting procedures, (3) provide informed consent, (4) willing to undergo rapid HIV and HCV testing and counselling, and (5) has positive urine analysis testing indicating current drug use. The RDS method was used to recruit study participants, a form of chain referral sampling designed to efficiently recruit a hidden population [11].

First, with the assistance of community health workers from a local community‐based organization, three initial participants were identified as seeds. Each participant recruited up to three PWID from their social network and received USD 11 (MYR 50) for their participation in the survey. If participants successfully recruited other PWID, they received an additional US$4.20 (MYR 20) for each PWID that they referred and who were eligible for the study. Sampling weights were corrected for selection bias, and network size was measured via self‐report and coupons. Second, trained interviewers administered the questionnaire in Bahasa Malaysia through face‐to‐face interaction or online interview (due to movement restriction during the COVID‐19 pandemic). Additional seeds were selected when enrolment was halted due to no new recruitments along the recruitment chain. The study has been approved by the Medical Research Ethics Committee, University of Malaya Medical Center and Medical Research (MRECID.NO: 202124–9804).

## 3 STUDY MEASURES/INSTRUMENTS

### 3.1 Variable selection

#### 3.1.1 The primary outcome of interest was HIV and HCV results

Rapid serological tests for HIV and HCV antibody were performed by community health workers, with a reactive test established that a person had been exposed to HIV/HCV virus.

#### 3.1.2 A range of variables were considered

We considered a range of variables including socio‐demographic characteristics: gender (man, woman, transwoman, transman), age (per 10 years older); ethnicity (Malay, Chinese, Indian, other); education; marital status; household income in MYR; and employment. Illicit drug use‐related variables selected included lifetime versus current drug use in the past 1 month, types of drugs and current mixing of drugs during injection (i.e. the act of physically combining or preparing multiple drugs together before use). Experiences with CJS‐related variables selected included lifetime and current experience in lock‐ups, prisons and compulsory drug detention centres (CDDCs). Lastly, access to health and social services‐related variables selected included lifetime and current access in the past 1 month to methadone services, needle‐syringe services and social services.

### 3.2 Data analysis

In estimating socio‐behavioural factors associated with HIV and HCV results, a generalized linear mixed‐effects model was constructed. Using an a priori‐defined modelling procedure, a full model that included all variables with *p*‐values less than 0.1 in bivariable analyses was fitted. After noting the Akaike Information Criterion (AIC) values for the full model, reduced model by using a backward selection approach, sequentially eliminating the variable with the largest *p*‐value, was constructed. The process was continued until zero variables were left in the model. Using this backward selection procedure, the final model with the best fit was selected, as indicated by the lowest AIC value.

## 4 RESULTS

Between September 2021 and March 2022, a number of 400 respondents were recruited. The majority of participants were men (94%), Malay (78%), had completed high school education (61%) and had full‐time/part‐time employment (66%). Among study participants, 22 (5.5%) individuals tested positive for HIV, while 162 (40.5%) participants tested positive for HCV. The weighted estimate of HIV prevalence in the sample is 5.03% (95% CI: 2.86−8.71). Meanwhile, the weighted estimate of HCV prevalence is 42.9% (95% CI: 35.5−50.6).

**Figure 1:**
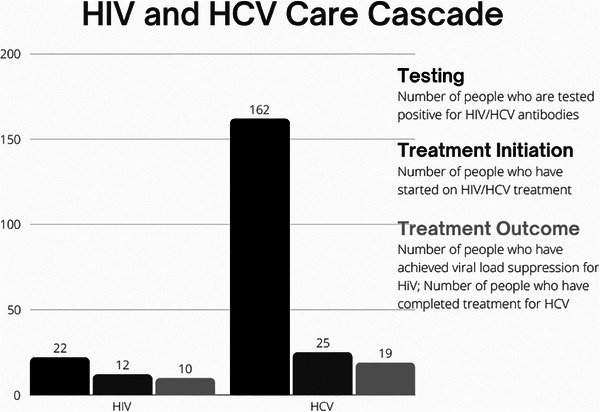
HIV and hepatitis C virus (HCV) care cascade among 400 people who inject drugs (PWID) in Klang Valley, Malaysia.

A number of 302 (75.5%) have previously tested for HIV, and of these, 277 (92%) were aware of their status. Among individuals who were HIV positive, 12 (55%) have started antiretroviral therapy (ART), and of these, 10 (83%) have achieved undetectable levels (<200 copies). Meanwhile, a number of 196 (49%) have previously tested for HCV, and of these, 174 (89%) were aware of their status. Among individuals who were HCV positive, only 25 (15%) have started HCV treatment, and of these, 19 (76%) completed treatment (Figure 1).

Table 1 provides information on socio‐demographic, drug use, exposure to the CJS and access to social and health services among participants who tested positive for HIV and HCV antibodies. Lifetime use of heroin and ATS, regardless of drug administration route, were reported among 392 (98%) and 370 (92.5%) individuals, respectively. Meanwhile, current use of heroin and ATS were reported among 340 (85%) and 328 (82%) individuals, respectively.

**Table 1:** Socio‐demographic factors among 400 HIV‐ and HCV‐positive PWID in Klang, Selangor

	*N* = 400	HIV positive *n* (%) *n* = 22	HIV negative *n* (%) *n* = 378	*p*‐value	HCV positive *n* (%) *n* = 162	HCV negative *n* (%) *n* = 238	*p*‐value
**Gender**							
Man	375 (93.8)	21 (95.5)	354 (93.7)	0.734	158 (97.5)	217 (91.2)	0.001
Woman	25 (6.2)	1 (4.5)	24 (6.3)		4 (2.5)	21 (8.8)	
**Age**							
<30	36 (9.0)	0 (0.0)	36 (9.5)	0.051	3 (1.9)	33 (13.9)	<0.001
31−40	127 (31.8)	3 (13.7)	124 (32.8)		30 (18.5)	97 (40.8)	
41−50	149 (37.2)	13 (59.0)	136 (36.0)		81 (50.0)	68 (28.5)	
>51	88 (22.0	6 (27.3)	82 (21.7)		48 (29.6)	40 (16.8)	
**Ethnicity**							
Non‐Malay	87 (21.8)	3 (13.6)	84 (22.2)	0.340	31 (19.1)	56 (23.5)	0.286
Malay	312 (78.0)	19 (86.4)	293 (77.5)		131 (55.0)	181 (76.1)	
**Education**							
< High school	244 (61.0)	13 (59.0)	231 (61.1)	0.850	90 (55.6)	154 (64.7)	0.066
High school or higher	156 (39.0)	9 (41.0)	147 (38.9)		72 (44.4)	84 (35.3)	
**Marital status**							
Never married	202 (50.5)	14 (63.6)	188 (49.7)	0.403	85 (52.5)	117 (49.1)	0.476
Married	81 (20.2)	4 (18.2)	77 (20.4)		28 (17.3)	53 (22.3)	
Widowed/separated	117 (29.3)	4 (18.2)	113 (29.9)		49 (30.2)	68 (28.6)	
**Household income (RM)**							
< 300	102 (25.5)	7 (31.8)	95 (25.1)	0.844	43 (26.5)	59 (24.8)	0.140
301−900	102 (25.5)	6 (27.3)	96 (25.4)		40 (24.7)	62 (26.1)	
901−1800	97 (24.3)	5 (22.7)	92 (24.4)		47 (29.0)	50 (21.0)	
> 1801	99 (24.7)	4 (18.2)	95 (25.1)		32 (19.8)	67 (28.1)	
**Employment**							
Unemployed	138 (34.5)	12 (54.5)	126 (33.3)	0.042	61 (37.7)	77 (32.4)	0.274
Full time/part time	262 (65.5)	10 (45.5)	252 (66.7)		101 (62.3)	161 (67.7)	
**Lifetime drug use (any route of administration)**							
Heroin	392 (98.0)	22 (100.0)	370 (97.9)	0.491	162 (100.0)	230 (96.6)	0.018
ATS	370 (92.5)	20 (90.9)	350 (92.6)	0.771	143 (88.3)	227 (95.4)	0.008
Cannabis	270 (67.5)	19 (86.4)	251 (66.4)	0.054	102 (60.7)	168 (70.6)	0.130
Kratom	159 (39.7)	13 (59.1)	146 (38.6)	0.062	59 (36.4)	100 (42.0)	0.239
**Current drug use in the past 1 month (any route of administration)**							
Heroin	340 (85.0)	21 (95.5)	319 (84.4)	0.232	150 (92.6)	190 (79.8)	0.007
ATS	328 (82.0)	14 (63.6)	314 (83.1)	0.021	124 (76.5)	204 (85.7)	0.019
Cannabis	79 (19.8)	6 (27.3)	73 (19.3)	0.826	23 (14.2)	56 (23.5)	0.065
Kratom	25 (6.3)	0 (0)	25 (6.6)	0.103	9 (5.6)	16 (6.7)	0.880
**Lifetime injection use**							
Heroin	371 (92.8)	22 (100.0)	346 (91.5)	0.155	153 (94.4)	215 (90.3)	0.137
ATS	98 (24.5)	5 (22.7)	87 (23.0)	0.975	38 (23.5)	54 (22.7)	0.858
**Current injection use in the past 1 month**							
Heroin	113 (28.3)	9 (40.9)	114 (30.2)	0.175	53 (32.7)	60 (25.2)	0.102
ATS	29 (7.3)	1 (4.5)	28 (7.4)	0.615	12 (7.4)	17 (7.1)	0.920
**Current mixing of drugs during injection in the past 1 month**	109 (27.3)	8 (36.4)	101 (26.7)	0.323	57 (35.2)	52 (21.8)	0.003
**Lifetime experience with the CJS**							
Lock‐ups	379 (94.8)	21 (95.5)	358 (94.7)	0.880	155 (95.7)	224 (94.1)	0.492
Prison	338 (84.5)	21 (95.5)	317 (83.9)	0.144	149 (92.0)	189 (79.4)	<0.001
CDDC	182 (45.5)	16 (72.7)	166 (43.9)	0.008	105 (64.8)	77 (32.4)	<0.001
**Current experience with the CJS in the past 1 month**							
Lock‐ups	32 (8.0)	0 (0)	32 (8.5)	0.155	15 (9.3)	17 (7.1)	0.155
Prison	28 (7.0)	0 (0)	28 (7.4)	0.593	10 (6.2)	18 (7.6)	0.593
CDDC	3 (0.8)	1 (4.5)	2 (7.4)	0.034	2 (1.2)	1 (0.4)	0.354
**Methadone services**							
Lifetime access	170 (42.5)	14 (63.6)	156 (41.3)	0.039	89 (54.9)	81 (34.0)	<0.001
Current access in the past 1 month	98 (24.5)	10 (45.5)	88 (23.3)	0.276	45 (27.8)	53 (22.3)	0.050
**Needle‐syringe services**							
Current access in the past 1 month	97 (24.3)	7 (31.8)	90 (23.8)	0.394	44 (24.7)	53 (22.3)	0.263
**Social services**							
Unmet need in the past 1 month	270 (67.5)	20 (90.9)	250 (66.1)	0.016	103 (63.6)	167 (70.2)	0.167

Abbreviations: ATS, amphetamine‐type stimulant; CDDC, compulsory drug detention centre; CJS, criminal justice system.

Among the study participants, 379 (94.8%), 338 (84.5%) and 82 (45.5%) reported to have entered lock‐ups, prisons and CDDCs, respectively. HCV antibody positivity was significantly higher among participants who had been in prison (92% vs. 79.4%, *p* < 0.001) or CDDC (64.8% vs. 32.4%, *p* < 0.001) compared to those who had not. Similarly, HIV positivity was higher among participants with a history of being in CDDC (72.7% vs. 43.9%, *p* = 0.008). Both participants who tested positive for HIV and HCV antibodies have a higher percentage of accessing lifetime methadone compared to participants who tested negative for HIV and HCV antibodies (63.6% vs. 41.3%, *p* = 0.039; 54.9% vs. 34%, *p* < 0.001, respectively). However, participants who tested negative for HIV and HCV antibodies have a low percentage of accessing methadone in the past 1 month (27.8% and 22.3%, *p* = 0.050).

Table 2 presents the results of bivariable and multivariable analysis of factors associated with positive HIV results among study participants. Adjusted analysis demonstrated that current use of ATS in the past 1 month was negatively associated with positive HIV results (Adjusted odds ratio [aOR] = 0.31, 95% CI: 0.12—0.86). Additionally, lifetime experience in CJS was associated with positive HIV results (aOR = 3.47, 95% CI: 1.02—14.7).

**Table 2:** Bivariable and multivariable analysis of factors associated with positive HIV results among 400 PWID in Klang, Selangor

	Unadjusted		Adjusted	
	Odds ratio (95% CI)	*p*‐value	Odds ratio (95% CI)	*p*‐value
Age (per 10 years)	1.30 (0.86–1.91)	0.198		
Ethnicity (non‐Malay vs. Malay)	1.82 (0.60–7.86)	0.346		
Gender (woman vs. man)	0.70 (0.04–3.59)	0.735		
Education (≥ higher vs. <high school)	1.08 (0.44–2.59)	0.850		
Marital status (married vs. non‐married)	0.68 (0.16–2.96)	0.595		
Household income (per RM100)	0.98 (0.94–1.01)	0.362		
Employment (yes vs. no)	0.42 (0.17–0.99)	0.048	0.52 (0.20–1.30)	0.160
Lifetime ATS use (yes vs. no)	0.80 (0.22–5.17)	0.771		
Lifetime marijuana use (yes vs. no)	3.18 (1.06–13.7)	0.067	3.27 (1.02–14.7)	0.072
Lifetime kratom use (yes vs. no)	2.26 (0.95–5.59)	0.068		
Current heroin use (yes vs. no)	3.88 (0.79–70.3)	0.189		
**Current ATS use (yes vs. no)**	**0.36 (0.15–0.93)**	**0.026**	**0.31 (0.12–0.86)**	**0.020**
Current mixing of injected drugs (yes vs. no)	1.57 (0.61–3.77)	0.327		
**Lifetime experience in CJS (yes vs. no)**	**4.01 (1.61–11.4)**	**0.005**	**3.47 (1.33–10.2)**	**0.015**
Lifetime access to methadone (yes vs. no)	2.49 (1.04–6.37	0.045	2.17 (0.86–5.80)	0.106
Current access to methadone (yes vs. no)	1.93 (0.62–7.28)	0.283		
Current access to NSP (yes vs. no)	1.49 (0.56–3.66)	0.397		
Unmet social service needs (yes vs. no)	5.12 (1.46–32.4)	0.029	4.34 (1.17–28.3)	0.058

The bold values statically significant <0.05.

Abbreviations: ATS, amphetamine‐type stimulant; CJS, criminal justice system; NSP, needle‐syringe programme.

Table 3 presents the results of bivariable and multivariable analysis of factors associated with positive HCV results among study participants. HCV‐positive results were associated with age (aOR = 1.58, 95% 1.22–2.08), gender (aOR = 0.28, 95% CI: 0.08–2.08) and education (aOR = 1.74, 95% CI: 1.08–2.82). Additionally, HCV‐positive results were associated with current heroin use (aOR = 2.44, 95% CI: 1.16–5.48), current mixing of drugs through injection use (aOR = 1.80, 95% CI: 1.08–3.03), past exposure to the CJS (lock‐ups, prison and CDDCs) (aOR = 3.32, 95% CI: 2.06–5.39), lifetime enrolment in methadone treatment (aOR = 2.30, 95% CI: 1.23–4.27) and current methadone treatment (aOR = 0.46, 95% CI: 0.23–0.92).

**Table 3:** Bivariable and multivariable analysis of factors associated with positive HCV results among 400 PWID in Klang, Selangor

	Unadjusted		Adjusted	
	Odds ratio (95% CI)	*p*‐value	Odds ratio (95% CI)	*p*‐value
**Age (per 10 years)**	**1.06 (1.04–1.09)**	**<0.001**	**1.58 (1.22–2.08)**	**<0.001**
Ethnicity (non‐Malay vs. Malay)	1.31 (0.80–2.16)	0.287		
**Gender (woman vs. man)**	**0.26 (0.08–0.70)**	**0.016**	**0.28 (0.08–0.84)**	**0.036**
**Education (≥ higher vs. <high school)**	**1.47 (0.98–2.21)**	**0.066**	**1.74 (1.08–2.82)**	**0.024**
Marital status (married vs. non‐married)	1.36 (0.76–2.47)	0.300		
Household income (per RM100)	1.00 (0.99–1.01)	0.698		
Employment (yes vs. no)	0.79 (0.52–1.20)	0.274		
Lifetime ATS use (yes vs. no)	0.36 (0.16–0.78)	0.010	0.42 (0.14–1.20)	0.108
Lifetime marijuana use (yes vs. no)	0.72 (0.47–1.10)	0.130		
Lifetime kratom use (yes vs. no)	0.78 (0.52–1.18)	0.239		
**Current heroin use (yes vs. no)**	**3.16 (1.67–6.42)**	**<0.001**	**2.44 (1.16–5.48)**	**0.023**
Current ATS use (yes vs. no)	0.54 (0.32–0.91)	0.020	0.85 (0.41–1.78)	0.656
**Current mixing of injected drugs (yes vs. no)**	**1.94 (1.24–3.04)**	**0.004**	**1.80 (1.08–3.03)**	**0.025**
**Lifetime experience in CJS (yes vs. no)**	**4.31 (2.83–6.64(**	**<0.001**	**3.32 (2.06–5.39)**	**<0.001**
**Lifetime access to methadone (yes vs. no)**	**2.36 (1.57–3.57)**	**<0.001**	**2.30 (1.23–4.27)**	**0.008**
**Current access to methadone (yes vs. no)**	**0.54 (0.29–1.00)**	**0.051**	**0.46 (0.23–0.92)**	**0.030**
Current access to NSP (yes vs. no)	1.30 (0.82–2.06)	0.263		
Unmet social service needs (yes vs. no)	0.74 (0.49–1.13)	0.168		

The bold values statically significant <0.05.

Abbreviations: ATS, amphetamine‐type stimulant; CJS, criminal justice system; NSP, needle‐syringe programme.

## 5 DISCUSSION

The present study observed a low prevalence of HIV (5.5%) among PWID in the study population. This is consistent with national surveillance data in Malaysia, which has shown a marked reduction in new HIV cases among PWID in the past decade [4]. In contrast, the prevalence of HCV was found to be high (41%) among PWID in our study population, however, is lower compared to previous local studies [8, 12, 13]. The decline in HIV prevalence among PWID may suggest higher mortality among HIV‐positive PWID, partly due to interruptions in ART. Structural barriers like stigma, criminalization and incarceration may limit continuous ART access, exacerbating poor health outcomes [14].

Age was found as a significant risk factor for HCV infection, although no such association was found for HIV. This finding underscores the need for targeted interventions for older PWID, who may have a longer duration of injecting drug use, increasing their cumulative exposure to HCV. Furthermore, older individuals may have engaged in riskier injection practices in the past [14, 15].

The number of participants currently injecting drugs is low, especially among ATS users. Injection cessation is prominent among study participants, likely due to their transition to methadone through harm reduction programmes and shifts in the drug market, such as changes in pricing and availability, which influence how drugs are consumed [4]. However, the use of heroin and ATS regardless of administrative routes remains high, exceeding 80%, reflecting the current local trend [16].

Participants with current heroin use were 2.44 times more likely to have HCV compared to those who did not. The sharing of needles is associated with HCV infection in two local studies [8, 12] and studies from the United States [17], with a longer duration of drug use was independently associated with HCV infection [18]. Our study also found that participants with current mixing of drugs through injection use were 1.8 times more likely to have HCV compared to those who did not. This finding suggests that the act of combining multiple substances for injection could significantly increase the risk of HCV transmission, potentially due to shared equipment or improper sterilization techniques [19].

Our analysis revealed that participants who tested positive for both HIV and HCV antibodies were more likely to have had lifetime access to methadone treatment compared to those who tested negative for both. This finding may reflect the longer history of injecting drug use and past exposure to unsafe injecting practices before initiating methadone treatment [20], especially among older PWID who are likely to engage with harm reduction programmes such as methadone. Additionally, lifetime access to methadone, as opposed to current use, may indicate discontinuation of treatment due to various factors, including disruptions caused by engagement with the CJS for drug‐related offenses [17, 21] This is also evident in our study in which both HIV‐ and HCV‐positive antibody results were significantly associated with experiences in the CJS. While these findings do not necessarily point to causation, they generally highlight that structural factors related to incarceration could promote risky drug use and disrupt services for PWID [19].

It is worth noting that our study had some limitations. First, the study was conducted in suburban areas of Klang Valley, Malaysia, and may not be representative of the entire country. Therefore, caution should be exercised when generalizing the findings to other regions. Second, the study relied on self‐reported data, which may be subject to recall and social desirability bias. Furthermore, this study measured HIV/HCV prevalence rather than incidence; therefore, findings do not establish causation. The lack of data on the timing of methadone initiation relative to CJS prevents us from determining whether methadone treatment preceded or followed incarceration. Future research should consider incorporating longitudinal designs to strengthen the validity of the findings. Third, we used RDS to generate our study population, which is highly dependent on accurate estimates of participants’ network sizes [22]. Inaccuracies in estimating network sizes can lead to overestimation or underestimation of outcome variables. However, a particular strength of RDS is its ability to enrol a diverse range of participants from different social networks [22].

## 6 CONCLUSIONS

In conclusion, our study provides valuable insights into the current prevalence of HIV and HCV among PWID in suburban areas of Klang Valley, Malaysia, highlighting distinct associations with different types of drug use. By providing comprehensive support and addressing the underlying factors that contribute to drug use including expanding access to methadone programmes, an enabling environment that promotes the health of PWID can be promoted. The findings underscore the importance of improving legal environments to reduce the transmission of these infections and improve the overall health outcomes of PWID.

## COMPETING INTERESTS

All authors declared no competing interests.

## AUTHORS’ CONTRIBUTIONS

NAMS, AK and DDJ designed the study. NAMS supervised the study. JP collected the data and managed the study database, and wrote the first draft of the manuscript. NAMS and MD conducted the primary statistical analysis. NAMS, VBK, AK and DDJ assisted in the interpretation of the data and provided intellectual input. All authors reviewed and approved the manuscript.

## FUNDING

This study is funded by the Institute of Research Management and Services, Universiti Malaya, University of Malaya's Impact‐Oriented Interdisciplinary Research Grant (IIRG005‐2020HWB). JP's doctoral study is supported by the Malaysian Implementation Science Training Program, funded by the US NIH Fogarty International Center (1D43TW011324‐01A1 ).

## DISCLAIMER

All respondents who participated in the study provided their informed consent.

## Data Availability

The full data that support the findings of this study are available from the corresponding author upon reasonable request.
